# Antioxidants Targeting Mitochondrial Oxidative Stress: Promising Neuroprotectants for Epilepsy

**DOI:** 10.1155/2020/6687185

**Published:** 2020-11-25

**Authors:** Nan Yang, Qi-Wen Guan, Fang-Hui Chen, Qin-Xuan Xia, Xi-Xi Yin, Hong-Hao Zhou, Xiao-Yuan Mao

**Affiliations:** ^1^Department of Clinical Pharmacology, Xiangya Hospital, Central South University, 87 Xiangya Road, Changsha 410008, China; ^2^Institute of Clinical Pharmacology, Central South University, Hunan Key Laboratory of Pharmacogenetics, 110 Xiangya Road, Changsha 410078, China; ^3^Engineering Research Center of Applied Technology of Pharmacogenomics, Ministry of Education, 110 Xiangya Road, Changsha 410078, China; ^4^National Clinical Research Center for Geriatric Disorders, 87 Xiangya Road, Changsha, 410008 Hunan, China; ^5^Department of Pharmacy, The First Affiliated Hospital of Gannan Medical University, Ganzhou, 341000 Jiangxi, China; ^6^Department of Pediatrics, Xiangya Hospital, Central South University, 87 Xiangya Road, Changsha, 410008 Hunan, China

## Abstract

Mitochondria are major sources of reactive oxygen species (ROS) within the cell and are especially vulnerable to oxidative stress. Oxidative damage to mitochondria results in disrupted mitochondrial function and cell death signaling, finally triggering diverse pathologies such as epilepsy, a common neurological disease characterized with aberrant electrical brain activity. Antioxidants are considered as promising neuroprotective strategies for epileptic condition via combating the deleterious effects of excessive ROS production in mitochondria. In this review, we provide a brief discussion of the role of mitochondrial oxidative stress in the pathophysiology of epilepsy and evidences that support neuroprotective roles of antioxidants targeting mitochondrial oxidative stress including mitochondria-targeted antioxidants, polyphenols, vitamins, thiols, and nuclear factor E2-related factor 2 (Nrf2) activators in epilepsy. We point out these antioxidative compounds as effectively protective approaches for improving prognosis. In addition, we specially propose that these antioxidants exert neuroprotection against epileptic impairment possibly by modulating cell death interactions, notably autophagy-apoptosis, and autophagy-ferroptosis crosstalk.

## 1. Overview

There are a plethora of investigations supporting that mitochondrial dysfunction forms an integral part of the development of neurological disorders and in particular epilepsy, a debilitating disease characterized by recurrent unprovoked seizures [1–4]. Evidence for the role of mitochondrial involvement in epilepsy arises from the knowledge that inherited mitochondrial disorders such as myoclonic epilepsy often appear. Mitochondria are prominent sources of reactive oxygen species (ROS) generation [5]. The release of ROS has deleterious effects on mitochondrial components such as mitochondrial DNA (mtDNA), mitochondrial membranes and respiratory chain proteins as well as nuclear DNA, leading to impaired mitochondrial function [6]. ROS overproduction-induced oxidative stress in mitochondria is emerging as a critical factor that is involved in the epileptogenesis and seizure generation. The direct evidence comes from studies in mice with deletion of mitochondrial antioxidant enzyme manganese superoxide dismutase 2 (Sod2 -/-) that exhibit spontaneous motor seizures [7], suggesting the contribution of oxidative stress-induced mitochondrial dysfunction to epileptic seizures. Additionally, enhanced activities of enzymes such as glutathione peroxidase (GPx) and glutathione reductase (GR) in mitochondria have also been demonstrated to exert neuroprotective effects against ROS-triggered oxidative damage in patients with epilepsy [8]. These data indicate that oxidative mitochondria damage can be diminished with clinical benefits by augmenting the activity of mitochondrial antioxidant enzymes. Enhancing antioxidant capacity of mitochondrial compartment may hold promise in disease prevention and treatment.

**Figure 1:**
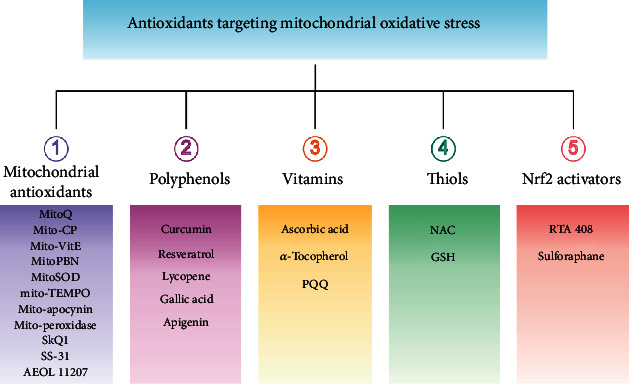
Classifications of antioxidants targeting mitochondrial oxidative stress. Generally, antioxidants targeting mitochondrial oxidative stress are divided into five different types including mitochondrial antioxidants, polyphenols, vitamins, thiols, and Nrf2 activators. The representative compounds are shown in each category. MitoQ: mitoquinolmesylate; Mito-CP: Mito-carboxy proxyl; SkQ1: visomitin; AEOL 11207: 5,15-bis(methoxycarbonyl)-10,20-bis-trifluoromethyl-porphyrinato manganese (III) chloride; PQQ: pyrroloquinoline quinone; NAC: N-acetylcysteine; GSH: glutathione; RTA-408: omaveloxolone; Nrf2: nuclear factor erythroid 2-related factor 2.

Antioxidants have the ability to counteract the deleterious effects of ROS and exert great benefits on oxidative stress-associated malformations including epilepsy. In recent years, large amounts of compounds that target mitochondria have been developed for diseases associated with impaired mitochondrial function via antagonizing mitochondrial oxidative stress (Figure 1) [9–14]. As summarized in Table 1, there are at least five different types of agents to counteract mitochondrial oxidative stress: mitochondrial antioxidants, polyphenols, vitamins, thiols, and nuclear factor E2-related factor 2 (Nrf2) activators. Substantial evidences have supported that antagonizing oxidative stress in mitochondria via antioxidants can attenuate or delay disease progression in a variety of experimental epilepsy models [15, 16]. Other antioxidants including melatonin, coenzyme Q10 (CoQ10), dimethyl malonate (DM), capsazepine (CPZ), glycyrrhizic acid (GA), and allopregnanolone (ALLO) also exhibit potent neuroprotection against epilepsy (details in aftermentioned sections).

Herein, our present review article aims to provide a discussion about the pathophysiology of mitochondrial oxidative stress in epilepsy and describe neuroprotection of antioxidants that mitigate mitochondrial oxidative stress against this disease. Furthermore, we also propose that modulation of interplay of distinct cell death modes is likely to be a critical mechanism for mitochondrially targeted antioxidants.

## 2. Mitochondrial Oxidative Stress and Epilepsy

### 2.1. Mitochondrial ROS Homeostasis

**Figure 2:**
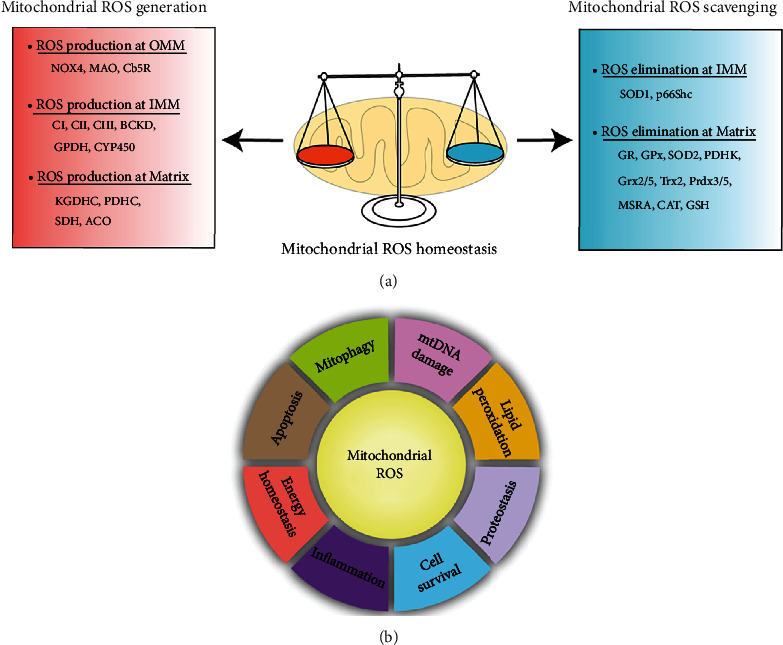
Homeostatic regulation and function of mitochondrial ROS (mtROS). (a) Under physical conditions, mtROS homeostasis is maintained by ROS generation (including ROS production at OMM, IMM, and matrix) and ROS scavenging (including ROS elimination at IMM and matrix) in mitochondria. (b) Mitochondrial ROS is implicated in multiple biological processes including mtDNA damage, mitophagy, apoptosis, energy homeostasis, inflammation, cell survival, proteostasis, and lipid peroxidation. OMM: outer mitochondrial membrane; NOX4: nicotinamide adenine dinucleotide phosphate oxidase subunit 4; MAO: monoamine oxidases; Cb5R: cytochrome b5 reductase; IMM: mitochondrial intermembrane space; CI: complex I; BCKD: branched-chain ketoacid dehydrogenase; GPDH: glycerol-3-phosphate dehydrogenase; CYP450: cytochrome P450; KGDHC: *α*-ketoglutarate dehydrogenase; PDHC: pyruvate dehydrogenase complex; SDH: succinate dehydrogenase; ACO: aconitase; SOD: superoxide dismutase; MSRA: methionine sulphoxide reductase A; CAT: catalase; GSH: glutathione; Grx2/5: glutaredoxin 2 and glutaredoxin 5; Trx2: thioredoxin 2; Prdx3/5: peroxiredoxin 3 and peroxiredoxin 5; GR: glutathione reductase; GPx: glutathione peroxidase; PDHK: pyruvate dehydrogenase kinase; ROS: reactive oxygen species.

Mitochondria are the prominent sites of ROS production within the cell, as large amounts of superoxide (O_2_^−^) are generated as byproducts of mitochondrial metabolism in many biochemical processes including electron escape from the electron transport chain (ETC) during mitochondrial oxidative phosphorylation (OXPHOS) and the tricarboxylic acid (TCA) cycle. It is generally accepted that the respiratory chain is the dominant ROS producer in mitochondria. Indeed, two of the respiratory chain complexes, namely complexes I (CI) and CIII, have been demonstrated, for a long time, to be involved in O_2_^−^ production [17, 18]. Mitochondrial ROS (mtROS) are also synthesized by several matrix proteins and complexes including enzymes of the TCA cycle (e.g., aconitase, pyruvate dehydrogenase, and *α*-ketoglutarate dehydrogenase) [19–22]. Additionally, numerous enzymatic reactions in mitochondria can also produce ROS, including those of glycerol-3-phosphate dehydrogenase, cytochrome P450, monoamine oxidase, and cytochrome b5 reductase. In fact, by their proximity to ROS, mitochondrial proteins, lipids and DNA are believed to be primary targets of oxidative damage during stress, creating a mitochondrial free radical “vicious cycle” of injury [6]. In addition to mtROS generation, homeostatic redox status in mitochondria is also controlled by mitochondria-associated antioxidant defenses (Figure 2). Accordingly, SOD2, peroxiredoxin 3 (Prdx3), GR, and thioredoxin 2 (Trx2) generally exert antioxidative role in mitochondria [23–26]. Physiologically, mtROS homeostasis is strictly maintained by free radical generation and scavenging systems. However, following the pathological conditions such as epilepsy, excessive ROS production appears, especially in the brain mitochondria, and results in oxidative damage.

### 2.2. Role of Mitochondrial Oxidative Stress in Epilepsy

*mtDNA damage*. Mitochondria have their own genome (mtDNA), which includes 37 genes and is maternally inherited. Following ROS stimuli, DNA can be damaged [27].In contrast to nuclear DNA (nDNA), mtDNA is more vulnerable due to lack of the protection of histones and the close proximity to electron transport chain, which is the predominant source of O_2_^−^ production [28]. The direct evidence arises from the fact showing that levels of oxidized bases in mtDNA are 2-3 folds more than nDNA [29]. It is well known that mtDNA encodes for vital subunits involved in OXPHOS by which mitochondria provides energy supply and mtROS balance is maintained. Oxidative mtDNA lesion hence contributes to defects in OXPHOS, which provides insufficient demands of a cell for energy and triggers excessive ROS production, thus creating a vicious cycle [30]. Oxidative damage to mtDNA, particularly those that affect ETC components, is suspected of contributing to onset and the progression of various human mitochondrial disorders [31]. The most common types of ROS-promoting mtDNA damage are point mutations due to oxidative modifications of purines and pyrimidines and also gene deletions. There are two major mutagenic consequences following ROS attack: the formation of 8-hydroxyguanine (8-OH-G) and structural impairment such as single and double strand breaks [32–34]. In the epilepsy research, ROS production from seizures leads to mtDNA damage and decreases activities of mtDNA-encoded mitochondrial ETC subunits, notably CI, CIII, CIV, and CV [35]. It indicates that the mtDNA is particularly vulnerable to oxidative lesion by ROS.

MtDNA damage can also be assessed by thymine glycol (TG) and 8-hydroxydeoxyguanosine (8-OHdG), an oxidative lesion of DNA [36, 37]. The ratio of steady state levels of 8-OHdG to TG often serves as a reliable index of oxidative DNA damage. Prior work demonstrates a time-dependent increase of 8-OHdG/2dG in mitochondria following kainic acid (KA-) induced epileptic seizures [38]. Similarly, these alterations are correlated with mitochondrial O_2_^−^ production accompanied by a transient decrease in mtDNA repair enzyme 8-oxoguanine glycosylase (Ogg1) [38].

*Mitophagy*. In 2016, Yoshinori Ohsumi was awarded the Nobel Prize in Physiology or Medicine for his work in the molecular machinery of autophagy. Mitophagy, a form of autophagy, selectively describes the selective removal of dysfunctional or superfluous mitochondria, which is vital for maintenance of mitochondrial homeostasis and cell survival. The best known regulators implicated in mitophagy are autophagy-related 32 protein (Atg32), NIP3-like protein X (NIX; also known as BNIP3L), parkin, and PTEN-induced putative kinase protein 1 (PINK1) [39]. NIX overexpression or downregulation of PINK1 results in induction of mitophagy via activation of mitochondrial depolarization [40, 41]. It has shown that mitophagy is activated by a variety of stressors including hypoxia, nutrient deficiency, and increased oxidative phosphorylation activity. In response to nerve growth factor deprivation, rapamycin, or starvation, mitophagy is specially triggered by ROS [42–44]. As noted above, mitochondria are considered as ROS-generating organelles. On the one hand, induction of mitophagy by mtROS under physical condition is beneficial for eliminating damaged mitochondria. On the other hand, excessive ROS-triggered oxidative stress due to mitochondrial dysfunction promotes altered mitophagy and leads to onset of multiple diseases. The brain is particularly susceptible to oxidative stress due to the high amount of oxygen consumption and lipid-rich content [45]. Thus, aberrant mitophagy (either uncontrollable mitophagy or inadequate mitophagy) due to mitochondrial defects via oxidative stress has a strong potential to cause neurological diseases such as epilepsy. Following neonatal seizures, it shows that mitophagy markers PINK, dynamin-related protein1 (Drp1), and polyhydroxybutyrate are significantly downregulated and treatment with leptin remarkably reverses abnormal mitophagy and seizure-associated cognitive deficits [46]. Defects in mitophagy are also observed in refractory temporal lobe epilepsy patients with hippocampal sclerosis as immunofluorescent analysis reveals colocalization of the autophagosome marker LC3B with the mitochondrial marker TOMM20 in hippocampi and temporal lobe cortexes [47]. Collectively, these results indicate that aberrant mitophagy due to mitochondrial dysfunction acts as important etiological factors of epilepsy.

*Apoptosis*. As we previously summarized, oxidative stress is a vital factor of activation of diverse cell death pathways including apoptosis [48]. Mitochondria play critical roles in apoptosis due to the high sensitivity to oxidative stress. It has shown that the opening of mitochondrial transition pore leads to the release of proapoptotic factors notable cytochrome c into the cytoplasm [49]. This process subsequently activates the caspase-dependent mitochondrial pathway, finally boosting apoptotic protease-activating factor-1 (Apaf-1) and procaspase-9 and facilitating the formation of the apoptosome [50]. Several factors are reported to be involved in modulating mitochondrial oxidative stress and cell apoptosis in epilepsy. During low-Mg^2+^-induced epileptiform activity, mitochondrial Ca^2+^ accumulation has the ability to stimulate the production of NADH and ROS [51]. Mitochondrial fission is also implicated in oxidative stress, apoptosis, and many neurological diseases. In pilocarpine-induced epilepsy rat model, mitochondrial fission is significantly increased and inhibition of mitochondrial fission protein Drp1 by mitochondrial division inhibitor 1 (mdivi-1) augments SOD activity, reduces expressions of cytochrome c and caspase-3 as well as increases neuron survival [52]. These results suggest the positive relationship between mitochondrial fission and onset of epileptic seizures, and inhibition of mitochondrial fission suppresses neuronal apoptosis via blocking mtROS/cytochrome c pathway. The mitochondria-localized receptor, NLRX1, was also previously found to protect against mitochondrial damage and epithelial cell apoptosis in an oxidative stress-dependent manner, as results from Stokman research group revealed that loss of NLRX1 led to increased oxidative stress and apoptosis in epithelial cells during ischemia-reperfusion injury [53]. It indicates the critical role of NLRX1 in the control of mitochondrial activity and prevention of oxidative stress and apoptosis in tissue injury, although whether NLRX1 mediates oxidative stress-associated apoptosis in the brain mitochondria remains to be elucidated following epileptic condition.

*Inflammation*. ROS and inflammatory response seem to interact with each other. It has shown that ROS-associated oxidative stress-induced mitochondrial dysfunction exacerbates inflammation in airway smooth muscle and cardiac tissue [54, 55]. Targeted scavenging of mitochondrial ROS by MitoVitE or MitoQ reduces inflammation and facilitates organ recovery [54, 55]. These results implicate a critical role of mitochondrial oxidative stress in the contribution to tissue inflammation. Following ROS burst in mitochondria, NACHT, LRR, and PYD domains-containing protein 3 (NLRP3) inflammasome can be activated and trigger the production of the proinflammatory cytokine IL-1*β*, leading to organ dysfunction [56]. Recovery of mitochondrial function suppresses ROS generation and reverses inflammatory response [56]. The ability of mtROS to activate NLRP3 activation may be the consequence of oxidative effects on mtDNA that exerts the inflammatogenic potential [57]. Additionally, NAD+/NADH redox imbalance induced by reduction of CI activity also facilitates NLRP3 inflammasome activation [58–60]. This suggests that mitochondrial oxidative damage promotes a pro-inflammatory state andcauses disease etipathogenesis. Although there is no direct evidence supporting the contribution of mitochondrial oxidative stress to inflammation in epilepsy, treatment with CoQ10 has evident neuroprotection against pentylenetetrazol- (PTZ-) induced kindling via suppressions of oxidative damage and neuroinflammation [61], which indirectly reflects the relationship between mitochondrial oxidative lesion and inflammation to a certain extent. Further investigation is essential to clarify this item.

## 3. Mitochondrially Targeted Antioxidants as Neuroprotective Agents for Epilepsy

As mentioned above, a large number of mitochondria-associated antioxidants have been developed. In this section, we focus on the compounds that are reported to exhibit neuroprotection against epilepsy.

### 3.1. Mitochondrial Antioxidants

In the past 40 years, diverse antioxidants, which directly target mitochondria, have been developed such as Mito-CP, Mito-TEMPO (SOD mimetic), Mito-peroxidase, MitoQ (ubiquinone), MitoPBN, MitoSOD, Mito-apocynin, Mito-VitE (vitamin E), SKQ1 (plastoquinone), SS-31, and AEOL 11207 [9–14]. Among these compounds, there are extensive publications of the neuroprotective effects of MitoQ against epileptic condition. It has demonstrated that treatment with MitoQ significantly rescues learning and memory deficits in pilocarpine-induced mice model of temporal lobe epilepsy via activation of cAMP response element-binding protein (CREB) [62]. The beneficial effect of MitoQ in the alleviation of memory decline is also found in Angelman syndrome [63], which is associated with epileptic pathology, and it is demonstrated that its therapeutic response is likely through reducing superoxide generation and suppressing neuronal apoptosis. These data point MitoQ as promising neuroprotective agents in epilepsy and associated comorbidities. In addition, the lipophilic metalloporphyrin, AEOL 11207, also exerts excellent anti-seizure property as a recent investigation has illustrated seizure behavior is observed in mice lacking mitochondrial SOD2 [7], and AEOL 11207 significantly alleviates this phenotype, which suggests the therapeutic potential of AEOL 11207 against epilepsy.

### 3.2. Polyphenols

Polyphenols are plant-based compounds that generally possess potent ROSscavenging properties. Extensive reports show that polyphenols including curcumin, resveratrol, and lycopene protect against epileptic brain damage via eliminating mitochondria-derived oxidative stress [64–66]. Curcumin protects mitochondria from ROS-induced lipid peroxidation and cell death, and exerts neuroprotective effects against epileptic seizures and cognitive deficits [64]. Additionally, resveratrol, which is extracted from the grape fruit, significantly decreases seizure-induced brain damage via reducing levels of O_2_^−^ and lipid peroxidation [65]. There is also evidence supporting that lycopene derived from tomato has the capacity to reverse seizure severity in PTZ-induced epileptic rodent model via reducing oxidative damage, notably decreased malondialdehyde (MDA) level and enhanced activities of CI, CII, and CIV [67]. Collectively, these results indicate that the naturally occurring compounds polyphenols show promise in the neuroprotection against epileptic pathology.

### 3.3. Vitamins

Dietary vitamins also display powerful ROS elimination ability and have been illustrated to possess neuroprotection against epilepsy. For instance, vitaminE (*α*-tocopherol and an analogue to vitamin E, trolox)has been shown to possess potent antioxidant and neuroprotective effects in rats with status epilepticus induced by pilocarpine [68] and the therapeutic response of agents is also shown in pediatric epilepsy [69]. The beneficial effect of this vitamin E is due to inhibition of CI activity and subsequently leads to suppression of mitochondrial oxidative stress in the brain of preclinical models [70]. Additionally, there are also indications showing that VitE suppresses mitochondrial oxidative stress in iron-induced neurotoxicity [71, 72]. These data implicate that VitE may serve as potential neuroprotectants against epilepsy.

### 3.4. Thiols

The thiols including glutathione (GSH) and N-acetylcysteine (NAC) are widespread compounds targeting mitochondrial oxidative stress. Extensive evidences describe that decreased levels of these molecules are observed in epilepsy and that increasing its neuronal concentrations alleviates epileptic damage [73–77]. As a type of peptide, GSH inactivates ROS directly and GR regenerates GSH from oxidized glutathione. Reduced GSH content is correlated to increased seizure frequency. NAC is considered as the acetylated precursor of GSH and shows powerful antioxidant activity, which is useful for the improvement of seizure severity and epileptogenesis [73]. Moreover, NAC also abrogates N-methyl-d-aspartate- (NMDA-) mediated excitotoxicity, which is associated with the pathogenesis of epilepsy and ROS generation [78]. Recently, the notion that NAC enhances the activities of SOD2 and Prdx3, two common antioxidant enzymes in mitochondria, suggests that it possesses strong antioxidative features associated to this organelle [79, 80].

### 3.5. Nrf2 Activators

Nrf2 is known to be a redox-sensitive transcription factor which induces antioxidant and detoxifying enzymes in order to protect cells from oxidative stress [81]. It has shown that Nrf2 activation is able to suppress mitochondrial oxidative stress and is beneficial for suppressing disease pathology including epilepsy [16]. For example, sulforaphane, a natural isothiocyanate, which is regarded as a common Nrf2 activator, has the capacity to combat oxidative damage in mitochondria and remarkably diminishes seizure threshold in pilocarpine-induced status epilepticus [82]. The protective effect of sulforaphane against epileptic seizures in mice may be associated with enhancing increased abilities of hippocampal mitochondria to generate ATP and suppressing lipid peroxidation [82]. These data imply that sulforaphane and/or Nrf2 activation are viable neuroprotective strategies to improve mitochondrial function through antioxidant mechanisms. RTA 408, another common Nrf2 activator [83], also blocks excessive ROS production and protects from neuronal death [16]. In the aspect of epilepsy field, RTA 408 is found to abrogate mtROS overproduction and reduces seizure frequency via activation of Nrf2 through inhibition of its cytoplasmic repressor Kelch-like ECH-associated protein 1 (KEAP1) [84]. Collectively, these results suggest that Nrf2 activators possibly exert neuroprotective effects against epilepsy via maintaining mitochondrial redox homeostasis.

### 3.6. Others

Apart from antioxidant agents, there are also other compounds which exhibit potent inhibition of mitochondrial oxidative stress and display neuroprotective potential against epilepsy. For instance, the endogenous antioxidant melatonin has been shown to be protective in human epilepsy, in the KA mice model and in the PTZ rat model [74, 85, 86]. The neuroprotection of melatonin against epileptic seizures may be linked with the abrogation of mitochondrial oxidative stress [74, 87]. Additionally, other components such as CoQ10, DM, CPZ, GA, and ALLO have demonstrated to exert strong antioxidative property, restore mitochondrial redox balance, and protect against epileptic brain damage [4, 24, 61, 75, 88].

## 4. Mitochondrial Oxidative Stress-Associated Antioxidants Are Proposed to Manipulate the Interplay of Cell Death Modes in Epilepsy

**Figure 3:**
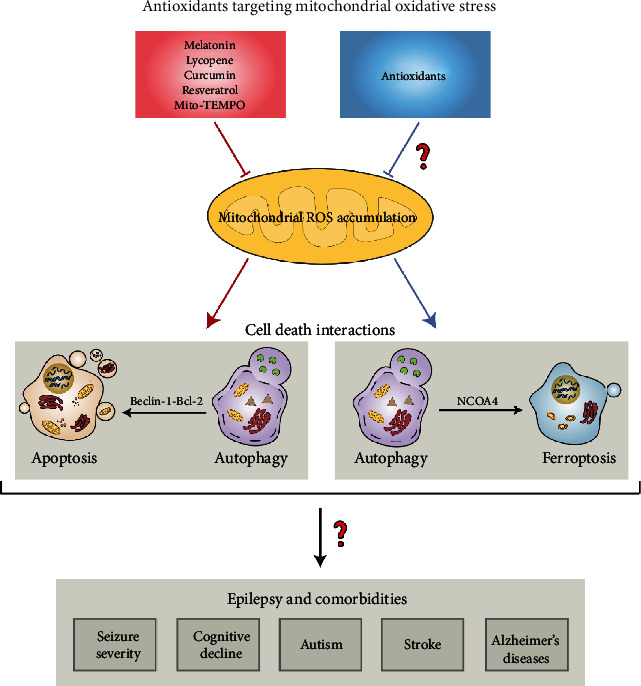
Antioxidants targeting mitochondria are proposed to modulate cell death interactions in epilepsy and comorbidities. Diverse antioxidants which counteract mitochondrial oxidative stress (melatonin, lycopene, curcumin, resveratrol, and Mito-TEMPO) are reported to regulate the interplay between autophagy and apoptosis and likely alleviate epilepsy and comorbidities (cognitive decline, autism, stroke, and Alzheimer's disease). As activation of autophagy often results in ferroptosis process, it is possibly that other antioxidants targeting mitochondria have the capacity to manipulate this intersection and finally protect brain from epileptic damage and associated complications. NCOA4: nuclear coactivator 4.

The interactions of various cell death modes have been discussed, since the identification of distinct types of cell death modalities. Recent investigations have depicted that antioxidants influence the crosstalk of apoptosis, autophagy, and ferroptosis, a novel type of regulated cell death, and modulate mitochondrial function. The interplay of these cell death modes is very complex. It is well known that at least two sorts of crosstalk of cell death types are affected by antioxidants: crosstalk between autophagy and apoptosis as well as crosstalk between autophagy and ferroptosis (Figure 3). The modulatory effect of antioxidative agent on the interplay of autophagy and apoptosis is linked with Beclin-1-Bcl-2 interaction while nuclear receptor coactivator 4 (NCOA4) is involved in the crosstalk between autophagy and ferroptosis. The polyphenol resveratrol blocks caspase-3-mediated apoptosis via suppressing autophagy in diabetic cardiomyopathy [89]. It suggests the contribution of autophagy to the induction of apoptosis. However, this notion is inconsistent with other work showing that the activation of autophagy by melatonin and lycopene abolishes apoptotic cascades [90, 91]. The discrepancy may be attributed to different action mechanisms of various types of antioxidants in mitochondria. In any way, these results indicate that mitochondrial oxidative stress-associated antioxidants have the potential to shape the interactions of autophagy and apoptosis. In the aspect of research on the coexistence of autophagy and ferroptosis, Mito-TEMPO-induced inhibition of autophagy is demonstrated to block ferroptosis process, suggesting a positive regulation of autophagic flux on ferroptotic cell death [92]. NCOA4-mediated ferrous iron accumulation often triggers ferroptosis via activation of autophagy (ferritinophagy). Treatment with Mito-TEMPO has the capacity to decrease GSH depletion and abrogate ferroptosis process and autophagic response. Collectively, these findings imply that diverse types of cell death modalities are possibly present within the cell and interact with each other, and antioxidants associated with mitochondrial oxidative stress may affect this network and possess neuroprotective potential against pathological conditions. In some cases, antioxidative agents are able to simultaneously manipulate different types of cell death demise. For instance, GA was previously found to protect juvenile epileptic rats against seizure-induced hippocampal damage via blocking neuronal apoptosis and promoting mitochondrial autophagy [75]. Taken together, it indicates that manipulation of diverse cell death interactions may serve as a critical neuroprotective mechanism of mitochondrial oxidative stress-linked antioxidants.

## 5. Limitations and Solutions of Antioxidants Targeting Mitochondria

### 5.1. Limitations

Despite promising neuroprotective potential of antioxidants against epileptic condition, there are still some limitations to be considered. These factors include bioavailability and blood-brain barrier (BBB) permeability [93]. The vast amounts of antioxidative agents display poor bioavailability and low BBB penetration [94, 95]. It is known that absorption and metabolism are key factors affecting the bioavailability of many molecules. It is possible to speculate that low bioavailability of antioxidant compounds is attributable to the poor absorption via oral administration route, rapid metabolism, and fast systematic elimination [96, 97]. Taking curcumin as an example, the poor absorption from gastrointestinal tract leads to low oral bioavailability with only 1% [98]. The low bioavailability of resveratrol is due to extensive hepatic phase II metabolism, which causes almost complete conversion into the conjugated metabolites and rapidly excreted the urinary tract [99]. With respect to BBB penetration, curcumin is found to have poor permeability through the BBB in a free form, which largely limits its biomedical application disease pathologies [93]. Taken together, future strategies are of urgent need to tackle the limitations and enhance the clinical utilization.

### 5.2. Strategies

Despite the existence of several flaws as described above for the antioxidant molecules, there are several alternatives to overcome the problem, which make them become promising neuroprotective agents. It has shown that chemical modification through the conjugation to a lipophilic cation, such as the triphenylphosphonium (TPP) cation, facilitates the bioavailability of antioxidants and its accumulation into the mitochondrial matrix, thereby protecting from mitochondrial oxidative damage [100]. Most of mitochondrial antioxidants such as MitoQ and Mito-VitE are obtained via this strategy. It has shown that Mito-VitE is 350-folds more potent than Trolox (water-soluble vitamin E analog) [101]. Besides, acetylation and esterification are also able to favor the bioavailability by reducing their autooxidation and enhance lipophilicity depending on uptake of cell [102–104]. Another alternative method that makes delivery of antioxidants is encapsulation technologies. Multiple antioxidants such as curcumin and resveratrol by encapsulation in nanoparticle exhibit favorable oral bioavailability and BBB-crossing property, finally enhancing delivery into the brain for neuroprotection and disease therapy [105, 106]. Recently, exosome-delivered strategy has been extensively utilized for disease therapy. Exosomes are defined as cell-derived bilayer lipid vesicles with a diameter between 40 and 100 nm, which has a lipid bimolecular structure and efficiently load hydrophobic and hydrophilic drugs. It has demonstrated that encapsulation of curcumin into the exosome significantly increases its bioavailability and triggers highly effective BBB crossing with no evident tissue toxicity, resulting in improvement of cognitive function in a mouse model of Alzheimer's disease [107]. These data implicate that it is possible to overcome the shortages of the antioxidants targeting mitochondrial oxidative stress in order to improve the neuroprotection against epilepsy.

## 6. Conclusions and Future Directions

The evidence discussed in the review supports that mitochondrial oxidative stress and dysfunction are implicated in the onset and progress of epilepsy. Antioxidants targeting mitochondrial oxidative stress have great neuroprotective effects against epileptic condition. Despite the detailed molecular mechanisms by which these molecules protect brain against epileptic seizures, many experimental studies have been conducted on the antioxidants that exhibit reliable seizure-suppressing properties. In fact, there are several mitochondria-associated antioxidants which are assessed for treating patients with epilepsy (Table 2). These data altogether conclude that the agents targeting mitochondrial oxidative stress hold promise as neuroprotectants against seizure generation and/or epileptogenesis.

There are also some items to be clarified [108, 109]. Firstly, although overwhelming investigations have revealed the association with mitochondrial oxidative damage and the pathogenesis of epilepsy, the regulatory mechanism of oxidation-mediated mitochondrial dysfunction following the pathological condition is complex, which requires further explorations in ongoing research. Clarification of this theme is beneficial for the development of novel antioxidants and other nonpharmacological interventions such as diet supplement and gene manipulation. Secondly, considerations of bioavailability and BBB penetration as mentioned above require more in-depth clinical trials, and the implementation of multicenter clinical investigation with large sample size is also essential to ascertain the beneficial effects of these antioxidants on the long-term consequence of epilepsy. Current clinical evaluation of the compounds is conducted to observe the outcomes of patients suffered from epilepsy with very limited sample size and merely in one hospital, which can not reflect the epidemiology. It is tricky to draw a reliable conclusion with the insufficient clinical sample due to genetic variation. Despite some mysteries remain to be clarified in the future, the antioxidative compounds targeted to mitochondria are undoubtedly favorable neuroprotective agents for improvement of brain damage in patients with epilepsy.

## Figures and Tables

**Table 1:** Neuroprotective effects of antioxidants in epileptic rodent models.

Category	Agent	Chemical structure	Model	Result	Reference
Mitochondrial antioxidants	MitoQ	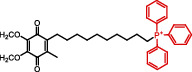	Pilocarpine injection	Memory deficits↓ Neuronal damage↓ pCREB↑	[17]
	AEOL 11207	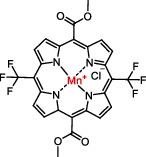	SOD2 (-/-) mice	Seizure frequency↓ Seizure duration↓ GSH↓ CII↑ Aconitase↑	[7]
Polyphenols	Curcumin	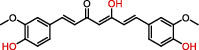	PTZ injection	Cognitive deficits↓ Cell loss↓ Lipid peroxidation↓ CI↑, CIV↑ GSH↑	[18]
	Resveratrol	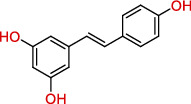	Litium-pilocarpine injection	Superoxide production↓ 4-HNE↓ CI↑	[19]
	Lycopene	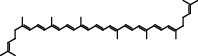	PTZ injection	Seizure score↓ CI↑, CII↑, CIV↑ GSH↑, SOD↑, CAT↑	[20]
Vitamins	Ascorbic acid	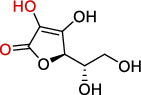	Kv1.1 (-/-) mice; KA injection	Seizure score↓ Mitochondrial respiration↑	[110]
	*α*-Tocopherol	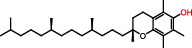	Kv1.1 (-/-) mice; KA injection	Seizure score↓ Mitochondrial respiration↑	[110]
Thiols	NAC	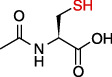	Audiogenic rats; 50 Hz stimulation	Seizure score↓ Seizure frequency↓ Cognitive deficits↓ Neuron loss↓ CAT↑ GSH↑	[73, 111]
Nrf2 activators	RTA 408	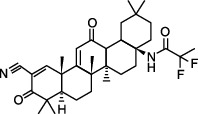	KA injection	Seizure frequency↓ Mitochondrial depolarization↓ Neuronal death↓ ROS accumulation↓ KEAP1↓ Nrf2↑	[16, 84]
	Sulforaphane	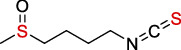	6 Hz stimulation; fluorothyl injection; PTZ injection; pilocarpine injection	Seizure latency↑ CI↑, CII↑ SOD↑, CAT↑	[25]
Others	Melatonin	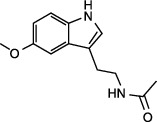	KA injection	Seizure severity↓ mtDNA damage↓ Lipid peroxidation↓ MDA↓ Seizure latency↑ SOD1↑, SOD2↑	[74, 112]
	CoQ10	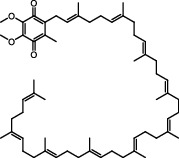	PTZ injection	Kindling score↓ MDA↓ CI↑, CII↑, CIV↑ GSH↑, SOD↑, CAT↑	[28]
	DM	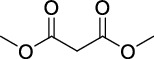	KA injection	Seizure severity↓ Neuronal degeneration↓ Mito-SOX↓	[4]
	GA	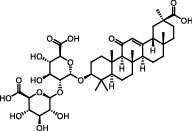	Litium-pilocarpine injection	Hippocampal damage↓ MDA↓ CIII↑ GSH↑, SOD↑	[29]
	ALLO	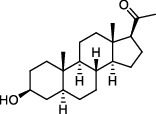	Pilocarpine injection	Cell death↓ mtDNA damage↓ ROS accumulation↓ SOD2↑	[30]

Note: MitoQ: mitoquinolmesylate; AEOL 11207: 5,15-bis(methoxycarbonyl)-10,20-bis-trifluoromethyl-porphyrinato manganese (III) chloride; NAC: N-acetylcysteine; RTA 408: omaveloxolone; CoQ10: coenzyme Q10; DM: dimethyl malonate; GA: glycyrrhizic acid; ALLO: allopregnanolone; PTZ: pentylenetetrazole; KA: kainic acid; GSH: glutathione; CI: complex I; CII: complex II; CIV: complex IV; MDA: malondialdehyde; 4-HNE: 4-hydroxynonenal; SOD: superoxide dismutase; CAT: catalase; Nrf2: nuclear factor erythroid 2-related factor 2; ROS: reactive oxygen species.

**Table 2:** Clinical evaluation of antioxidants in patients with epilepsy.

Identifier	Agent	Study subject	Status
NCT02369822	Vitamin C	Patients between 2 and 16 years (*n* = 60)	Unknown
NCT04488172	Vitamin E	Patients between 20 and 65 years (*n* = 200)	Recruiting
NCT00004637	Vitamin E	Patients between 1 and 18 years (*n* = 50)	IV
NCT00965575	Melatonin	Patients between 6 and 11 years (*n* = 10)	II
NCT01161108	Melatonin	Patients between 5 and 17 years (*n* = 13)	III
NCT02195661	Melatonin	Patients between 0.5 and 13 years (*n* = 193)	III
NCT03590197	Melatonin	Patients between 18 and 60 years (*n* = 104)	IV
NCT01370486	Melatonin	Patients between 18 and 55 years (*n* = 6)	Unknown
NCT04488172	Coenzyme Q10	Patients between 20 and 65 years (*n* = 200)	Recruiting
NCT00044252	Allopregnanolone	Patients between 18 and 45 years (*n* = 50)	Completed
NCT01673828	Allopregnanolone	Patients between 16 and 65 years (*n* = 13)	II

## Abbreviations

ROS: Reactive oxygen species
mtDNA: Mitochondrial DNA
SOD2: Manganese superoxide dismutase 2
GPx: Glutathione peroxidase
GR: Glutathione reductase
Nrf2: Nuclear factor E2-related factor 2
CoQ10: Coenzyme Q10
DM: Dimethyl malonate
CPZ: Capsazepine
GA: Glycyrrhizic acid
MitoQ: Mitoquinolmesylate
Mito-CP: Mito-carboxy proxyl
SkQ1: Visomitin
AEOL 11207: 5,15-Bis(methoxycarbonyl)-10,20-bis-trifluoromethyl-porphyrinato manganese (III) chloride
PQQ: Pyrroloquinoline quinone
NAC: N-Acetylcysteine
GSH: Glutathione
RTA-408: Omaveloxolone
ALLO: Allopregnanolone
PTZ: Pentylenetetrazol
KA: Kainic acid
CI: Complex I
CII: Complex II
CIV: Complex IV
MDA: Malondialdehyde
4-HNE: 4-Hydroxynonenal
SOD: Superoxide dismutase
CAT: Catalase
O_2_^−^: Superoxide
ETC: Electron transport chain
OXPHOS: Oxidative phosphorylation
TCA: Tricarboxylic acid
mtROS: Mitchondrial ROS
Prdx3: Peroxiredoxin 3
Trx2: Thioredoxin 2
OMM: Outer mitochondrial membrane
NOX4: Nicotinamide adenine dinucleotide phosphate oxidase subunit 4
MAO: Monoamine oxidases
Cb5R: Cytochrome b5 reductase
IMM: Mitochondrial intermembrane space
BCKD: Branched-chain ketoacid dehydrogenase
GPDH: Glycerol-3-phosphate dehydrogenase
CYP450: Cytochrome P450
KGDHC: *α*-Ketoglutarate dehydrogenase
PDHC: Pyruvate dehydrogenase complex
SDH: Succinate dehydrogenase
ACO: Aconitase
SOD: Superoxide dismutase
MSRA: Methionine sulphoxide reductase A
Grx2/5: Glutaredoxin 2 and glutaredoxin 5
Prdx3/5: Peroxiredoxin 3 and peroxiredoxin 5
PDHK: Pyruvate dehydrogenase kinase
nDNA: Nuclear DNA
8-OH-G: 8-Hydroxyguanine
8-OHdG: 8-Hydroxydeoxyguanosine
Ogg1: 8-Oxoguanine glycosylase
Atg32: Autophagy-related 32 protein
NIX: NIP3-like protein X
BNIP3L: NIP3-like protein X
PINK1: PTEN-induced putative kinase protein 1
Drp1: Dynamin-related protein1
Apaf-1: Apoptotic protease-activating factor-1
mdivi-1: Mitochondrial division inhibitor 1
NLRP3: NACHT, LRR, and PYD domain-containing protein 3
CREB: cAMP response element-binding protein
NMDA: N-Methyl-d-aspartate
KEAP1: Kelch-like ECH-associated protein 1
NCOA4: Nuclear receptor coactivator 4
BBB: Blood-brain barrier
TPP: Triphenylphosphonium.
